# Engineered extracellular vesicles as versatile ribonucleoprotein delivery vehicles for efficient and safe CRISPR genome editing

**DOI:** 10.1002/jev2.12076

**Published:** 2021-03-16

**Authors:** Xingang Yao, Pin Lyu, Kyung Yoo, Manish Kumar Yadav, Ravi Singh, Anthony Atala, Baisong Lu

**Affiliations:** ^1^ Wake Forest Institute for Regenerative Medicine Wake Forest University Health Sciences Winston‐Salem North Carolina USA; ^2^ State Key Laboratory of Organ Failure Research Guangdong Provincial Key Laboratory of New Drug Screening School of Pharmaceutical Sciences Southern Medical University Guangzhou P. R. China; ^3^ Department of Cancer Biology Wake Forest University Health Sciences Winston‐Salem North Carolina USA

**Keywords:** adenine base editor, aptamer, aptamer‐binding protein, CD63, CRISPR/Cas9, delivery, extracellular vesicle, ribonucleoprotein

## Abstract

Transient delivery of CRISPR‐based genome editing effectors is important to reduce off‐target effects and immune responses. Recently extracellular vesicles (EVs) have been explored for Cas9 ribonucleoprotein (RNP) delivery. However, lack of mechanisms to enrich RNPs into EVs limited the efficiency of EVs as a RNP delivery vehicle. Here we describe a mechanism to actively enrich RNPs into EVs. We used the specific interaction between RNA aptamer and aptamer‐binding protein (ABP) to enrich RNPs into EVs. We inserted RNA aptamer com into single guide RNA (sgRNA), and fused com‐binding ABP Com to both termini of tetraspan protein CD63 that is abundant in exosomes. We found that the Com/com interaction enriched Cas9 and adenine base editor (ABE) RNPs into EVs, via forming a three‐component complex including CD63‐Com fusion protein, com‐modified sgRNA and Cas9 or ABE. The RNP enriched EVs are efficient in genome editing and transiently expressed. The system is capable of delivering RNPs targeting multiple loci for multiplex genome editing. In addition, Cas9 from different species can be used together. The EV‐delivered RNPs are active in vivo. The data show that the aptamer and ABP interactions can be utilized to actively enrich RNPs into EVs for improved genome editing efficiency and safety.

## 1 INTRODUCTION

Extracellular vesicles (EVs) can be broadly divided into two main categories, exosomes and microvesicles, based on the mechanisms of generation. Exosomes are heterogeneous membranous vesicles released by various cells via inward budding of multivesicular bodies and subsequent fusion of the multivesicular body membranes with the plasma membrane (Heijnen et al., 1999). Exosomes play important roles in intercellular crosstalk and disease pathogenesis, and are believed to function by transporting RNAs, proteins and lipids from one cell to the other (Ratajczak et al., 2006). Microvesicles are generated by the outward budding and fission of the plasma membrane and the subsequent release of vesicles into the extracellular space. Exosomes and microvesicles overlap in sizes and currently it is difficult to separate the two types of vesicles in preparation.

The transportation capability of EVs prompted the exploration of using EVs as drug delivery vehicles (Alvarez‐Erviti et al., 2011; Yoo et al., 2018). Recently EVs are explored as vehicles to deliver Cas9 RNPs (Campbell et al., 2019; Chen et al., 2019; Gee et al., 2020; Kim et al., 2017; Montagna et al., 2018; Ye et al., 2020) or dCas9 RNPs (Lainscek et al., 2018) for gene editing and gene regulation purposes. All of these studies did not have a mechanism to actively enrich RNPs into EVs (Campbell et al., 2019; Chen et al., 2019; Gee et al., 2020; Kim et al., 2017; Lainscek et al., 2018; Montagna et al., 2018), except in one study that antigen/antibody interaction was used to enrich RNPs into exosomes and relatively low gene editing activity was reported (Ye et al., 2020). Mechanisms to actively enrich RNPs into EVs are needed to improve gene editing efficiency using EV as a delivery vehicle.

Recently we used the specific interactions between aptamer and aptamer‐binding proteins (ABPs) to package Cas9 (Lu et al., 2021; Lyu et al., 2019, 2020) or adenine base editor RNPs (Lyu et al., 2021) into lentiviral capsids for efficient delivery of RNPs into human cells (reviewed in (Lyu et al., 2020)). We inserted short RNA aptamer com into sgRNA scaffold and fused ABP Com with nucleocapsid protein of the lentiviral Gag protein. The specific interaction between com and Com recruits sgRNA into the lentiviral capsids, while the intrinsic affinity between sgRNA and Cas9 protein mediates the packaging of the whole RNP complex into lentiviral capsids. These capsids are highly efficient in gene editing and generated > 80% INDEL rates on multiple targets.

Tetraspan membrane protein CD63 is enriched in exosomes and is often used as a molecular marker for exosomes (Escola et al., 1998; Kobayashi et al., 2000). It has been used as a fusion partner of ABP for enriching aptamer‐containing mRNAs into EVs (Hung & Leonard, 2016). However, the EV delivered mRNAs were not functional due to quick degradation in recipient cells (Hung & Leonard, 2016). Vesicular stomatitis virus G protein (VSV‐G) is essential for vesicular stomatitis virus (VSV) infection by helping the viral particles to escape from the endosomes via low pH induced membrane fusion (Florkiewicz & Rose, 1984). This fusogenic activity makes VSV‐G a widely used pseudotyping envelope protein for lentiviral vectors (Akkina et al., 1996; Naldini et al., 1996; Reiser et al., 1996), and a fusogenic protein for membrane and protein delivery (Mangeot et al., 2011; Yang et al., 2017).

We reasoned that mechanisms for recruiting Cas9 RNPs into EVs and helping the RNPs to escape from the endosome system after delivery will greatly increase the efficiency of EV mediated RNP delivery. Here we describe a method to actively recruit Cas9 or ABE RNPs into EVs. We fused CD63 with ABP Com and used the com/Com interaction to actively enrich Cas9 RNPs into EVs. We further used VSV‐G protein to help the escape of Cas9 RNPs from the endosome system in recipient cells. Our data show that the engineered EVs show improved efficiency and safety as a RNP delivery vehicle for genome editing.

## 2 MATERIALS AND METHODS

### 2.1 Plasmids

CD63‐pEGFP‐C2 (Addgene #62964) and pMD2.G (Addgene #12259) were purchased from Addgene. Plasmids generated by this group are described in Table S1. They will be made available through Addgene (Addgene plasmid No. 138350) or upon request. Gene synthesis was done by GenScript Inc. All constructs generated were confirmed by Sanger sequencing. Sequence information for primers and oligoes was in Table S2. Target sequences for sgRNAs were listed in Table S3.

### 2.2 GFP reporter assays for gene editing activities

HEK293T‐derived *HBB‐IL2RG* EGFP reporter cells with target sequences for human beta haemoglobin (*HBB*) sickle cell mutant and human *IL2RG* (Javidi‐Parsijani et al., 2017), and *DMD* reporter cells with target sequence from human *DMD* exon 53 (Lyu et al., 2020) were used to detect gene editing activities of Cas9 RNPs targeting *HBB/ IL2RG* and *DMD* exon 53 respectively. The GFP‐reporter cells expressed no EGFP due to the disruption of the EGFP reading frame by the insertion of the respective target sequences right after the start codon of EGFP coding sequence. INDELs formed after gene editing may restore the EGFP reading frame, resulting in EGFP expression. GFP‐positive cells were analyzed by fluorescence microscopy or flow cytometry as detailed below.

Flow cytometry was performed with the Accuri C6 system of BD Biosciences as described (Javidi‐Parsijani et al., 2017; Lu et al., 2019; Lyu et al., 2020). Single cell suspension was made in PBS/0.5% FBS for analysis. The reporter cells without gene editing (and thus without fluorescent protein expression) were used as negative controls and a marker was placed at the position so that 99.9% of the cells were on the left side of the marker (fluorescence negative). In treated samples, cells on the right side of the marker were considered positive (see Figure S1 for representative data).

### 2.3 Production of Cas9 or ABE RNP‐enriched extracellular vesicles (EVs)

RNP‐enriched EVs were produced by co‐transfection of three plasmids into HEK293T cells: plasmid DNA expressing a fusion protein between ABP Com and CD63, pDM2.G expressing VSV‐G, and the target plasmid expressing the gene editing effector (SaCas9, SpCas9 or ABE) and the respective gene‐specific single guide RNA, sgRNA (See Table S1 for various target plasmids). Briefly, 5 million actively proliferating HEK293T cells grown in 10‐cm dishes were incubated with 5 ml Opti‐MEM. A total of 3 μg of Com‐CD63‐Com fusion protein expressing plasmid, 3 μg pMD2.G and 12 μg target plasmid DNA were mixed in 0.5 ml Opti‐MEM. Fifty‐four μl of Fugene HD (Promega) or 54 μg of polyethylenimine (Synchembio, Cat # SH‐35421) were mixed in 0.5 ml Opti‐MEM. The DNA mixture and the transfection reagent mixture were then mixed and incubated at room temperature for 15 min before they were added to the cells in Opti‐MEM. 24 h after transfection, the medium was changed to 10 ml Opti‐MEM and the RNP‐enriched EVs were collected 72 h after transfection. For transfection of cells grown in other tissue culture vessels, the amounts of DNA and transfection reagent were scaled based on tissue culture surface area.

### 2.4 EV concentration

Ultracentrifugation was used to concentrate EVs from tissue culture medium following our published procedures (Lu et al., 2017). Briefly, the cell culture medium was centrifuged at 1000 × *g* for 30 min at 4°C to remove cell debris. The supernatant was centrifuged at 120,000 × *g* for 70 min at 4°C. The pellet was washed once with PBS and centrifuged again under the same conditions. The resulting pellet containing the EVs was resuspended in PBS. Typically, EVs from 10 ml supernatants were resuspended in 500 μl (20x concentration) for in vitro experiments. These EVs can be stored at ‐80°C or be used immediately. It is better not to freeze and thaw the EVs more than twice.

### 2.5 Nanoparticle tracking analysis of EVs

Hydrodynamic diameters and concentrations of EVs were measured using the Nanosight NS500 instrument (Malvern Instruments, UK) using the instrument's software (version NTA3.2). The instrument was primed using phosphate buffered saline (PBS), pH 7.4 and the temperature was maintained at 25°C. Accurate particle tracking was verified using 50 nm and 100 nm polystyrene nanoparticle standards (Malvern Instruments) prior to examining samples. Concentrated samples containing EVs were serial diluted 1000 fold in PBS. The linear range for quantification of EV concentration in each sample fell between 10–40,000 fold dilutions. Therefore, all samples were diluted 1000‐fold in PBS. Five independent measurements (60 s each) were obtained for each sample in triplicate. Data are reported as the mean (multiplied by dilution factor for concentration determination) of these measurements ± standard error of the mean.

### 2.6 EV mediated RNP delivery

RNP enriched EVs concentrated from supernatant of 0.6∼20 million cells were added to 2.5 × 10^4^ cells grown in Opti‐MEM (ThermoFisher, Cat# 11058021) in 24‐well plates. It is important that at this time the medium has low serum since the presence of FBS in the medium inhibits EV mediated RNP delivery. After incubation for 12–24 h, the medium was changed to DMEM medium with 10% FBS. Thirty‐six hours after EV treatment, gene editing was analyzed by flow cytometry, fluorescent microscopy or Next‐generation sequencing (NGS) to be detailed later.

### 2.7 Examining degradation of EVs delivered Cas9

HEK293T cells (2.5 × 10^4^) were grown RMPI1640 medium with 0.5% FBS in 24 well plate to limit proliferation. The cells were treated with RNP‐loaded EVs at different time but were collected at the same time. EVs secreted by 0.2 million cells in 48 h (∼8 × 10^9^ vesicles) were added to each well. Just before EV treatment, the medium was changed to Opti‐MEM medium. The cells were collected 6 h, 12 h, 18 h, 24 h, 36 h and 48 h after EV addition, washed twice with PBS buffer, and lysed in laemmli buffer for western blotting analysis.

### 2.8 Western blotting analysis of EVs

EVs secreted by 5 × 10^6^ cells in 48 h were concentrated and resuspended in 500 μl PBS. Protein concentration was determined with the Pierce BCA Protein Assay Kit (Pierce ECL western blotting substrate, Cat.32106). Sixty μl of EV solution were mixed with equal volume of 2x laemmli buffer, boiled at 95 °C for 5 min. Ten μl of each sample were loaded in each lane of a 10% SDS‐PAGE gel for Western blotting analysis. The antibodies used include mouse monoclonal anti‐SaCas9 antibody (Millipore Sigma, Cat. MAB131872, clone 6F7, 1:1000), SpCas9 antibody (ThermoFisher, Cat. MA1‐201, 7A9‐3A3, 1:1000), GAPDH antibody (CST, 1:1000), RAB5B antibody (Abcam, Cat. ab230020, 1:1000), CD9 antibody (SBI, Cat. EXOAB‐CD9A‐1, 1:1000), VSV‐G antibody (Sigma, Cat. V4888, 1:1000), CD63 antibody (Abcam, Cat. ab68418, 1:1000), GRP94 antibody (CST, Cat. 20292T, 1:1000), HRP conjugated anti‐rabbit IgG (H + L) (ThermoFisher, Cat No. 31460, 1:5000) secondary antibody and anti‐mouse IgG (H + L) secondary antibody (ThermoFisher, Cat # 31430, 1:5000) in Western blotting. Purified SaCas9 protein (Biovision, M1280‐50‐1) and SpCas9 protein (GenScript, Z03389S) were used as standards in Western blotting to estimate protein amount. Chemiluminescent reagents (Pierce ECL western blotting substrate, Cat.32106) were used to visualize the protein signals under the LAS‐3000 system (Fujifilm). Protein amount was quantitated by densitometry analysis of Western blotting images by NIH ImageJ.

### 2.9 RT‐qPCR and qPCR analyses

A RNeasy Plus Mini Kit (QIAGEN Cat No. 74136) was used to isolate RNA from collected EVs. The QuantiTect Reverse Transcription Kit (QIAGEN) was used to reverse‐transcribe the RNA to cDNA. For *IL2RG sgRNA1* and *IL2RG sgRNA1^Tetra‐com^* detection, Scid‐g2F and sgRNA‐R3 were used as primers in SYBRGreen based RT‐qPCR. ABE‐g5‐onF and g5‐ABE‐R were used as qPCR primers to detect base editing at site 5. PCR was run on an ABI 7500 instrument. Primer information was included in Table S2.

### 2.10 Transmission electron microscopy

Transmission electron microscopy was performed at the Cellular Imaging Shared Resource of Wake Forest Baptist Health Center (Winston‐Salem, NC). EVs were stained with uranyl acetate for transmission electron microscopy. A Carbon grid (Ted Pella, CA) was immersed into 50 μl of concentrated EVs (about 6.0 × 10^11^ vesicles/ml). Excess sample was wicked from the grid leaving a wet film. One drop of 2% aqueous uranyl acetate was applied to the grid for 1 min. Excess stain was wicked from the grid with filter paper. Grid was allowed to dry while being held by anti‐capillary forceps and then placed on filter paper with the sample side up. The samples were observed under a FEI Tecnai G2 30 electron microscope and images were captured using 80 kV (FEI, Hillsboro, OR). The diameters of the particles were measured with NIH ImageJ software (Version 1.49).

### 2.11 Next‐generation sequencing (NGS) and data analysis

Genomic DNA was isolated from cultured cells with the DNeasy Blood & Tissue Kit (Qiagen). The DNA region containing the target sequences were amplified by the proofreading HotStart ReadyMix from KAPA Biosystems (Wilmington, MA). PCR primers used for amplifying each target sequence were listed in Table S2. The purified PCR products were shipped to Genewiz Inc. (Morrisville, NC) to perform next generation sequencing using the Amplicon EZ service. Usually 50,000 reads/amplicon were obtained. Analysis of insertions and deletions (INDEL) was done with the online Cas‐Analyzer software (Park et al., 2017) and CRISPRESSO2 (Clement et al., 2019), which gave very similar results.

### 2.12 Animals

The del52hDMD/mdx mice have been described recently and are kind gifts from Annemieke Aartsma‐Rus and Maaike van Putten (Veltrop et al., 2018). These mice carry copies of human *DMD* gene with exon 52 deletion in an *mdx*, C57BL/6 background. After arrival the mice were housed in the pathogen‐free animal facility at Wake Forest University Health Sciences. Experiments were conducted in accordance with the National Research Council Publication Guide for Care and Use of Laboratory Animals, and approved by the Institutional Animal Care and Use Committee of Wake Forest University Health Sciences (Animal protocol number A18‐087). Mice were kept in microisolator cages with 12‐h light/dark cycles and were fed ad libitum. Carbon dioxide (CO_2_) overdose, which causes rapid unconsciousness followed by death, was used to euthanize mice. The mice were exposed to CO_2_ without being removed from their home cage, so that the animals were not stressed by handling or being moved to a new environment. The CO₂ flow rate was set to displace 10% to 30% of the cage volume per minute. When the mice showed deep narcosis, they were subjected to cervical dislocation as a secondary method of euthanasia. After euthanasia, muscle tissues were collected for further analyses.

### 2.13 Intramuscular injection of RNP loaded EVs to mouse tibialis anterior (TA) muscle

Female del52hDMD/*mdx* mice of 5 months old were used for injection. EVs secreted by 5 million cells (low dosage) or 40 million cells (high dosage) in 48 h were injected into each TA muscle using a HAMILTON syringe. The mice were sacrificed 7 days after injection. TA muscles were removed for cryosections. The tissues were mounted in OCT embedding compound and frozen in liquid nitrogen. Six or seven blocks of three continuous 10‐μm‐thick sections, 200 μm away from each block, were collected for histological analysis. The rest tissues were used to purify genomic DNA for NGS analysis.

### 2.14 Immunohistochemistry assay

The sections were fixed in 4% paraformaldehyde for 10 min. After three times of PBS washing, the sections were incubated with 0.2% Triton X‐100/PBS buffer for 10 min, and with Protein Block (Agilent, Cat. X090930‐2) for 30 min. The sections were then incubated with rabbit anti‐dystrophin primary antibody (Abcam ab15277, 1:1000) for 1 h. Then the Alexa fluor 633 anti‐rabbit secondary antibodies (ThermoFisher, A‐21052, 1:5000) was incubated with the tissues for 45 min after PBS washing for three times. Then the slides were washed six times with PBS and mounted in DAPI‐containing mounting medium (ThermoFisher, D1306) for 5 min. The slides were observed under a fluorescence microscope.

### 2.15 Statistical analysis

GraphPad Prism software (version 5.0) was used for statistical analyses. T‐tests were used to compare the averages of two groups. Analysis of Variance (ANOVA) was performed followed by Tukey post hoc tests to analyze data from more than two groups. *P* < 0.05 was regarded as statistically significant.

## 3 RESULTS

### 3.1 CD63, aptamer and ABP mediated RNP enrichment in extracellular vesicles (EVs)

Current systems using EVs to deliver Cas9 RNPs do not have mechanisms to actively enrich RNPs into EVs (Campbell et al., 2019; Chen et al., 2019; Gee et al., 2020; Kim et al., 2017; Lainscek et al., 2018; Montagna et al., 2018) or have unsatisfactory efficiency (Ye et al., 2020). We set out to design a novel strategy to enrich RNPs into EVs through associating the RNPs with CD63, a protein enriched in exosomes (Escola et al., 1998; Kobayashi et al., 2000).

**FIGURE 1:**
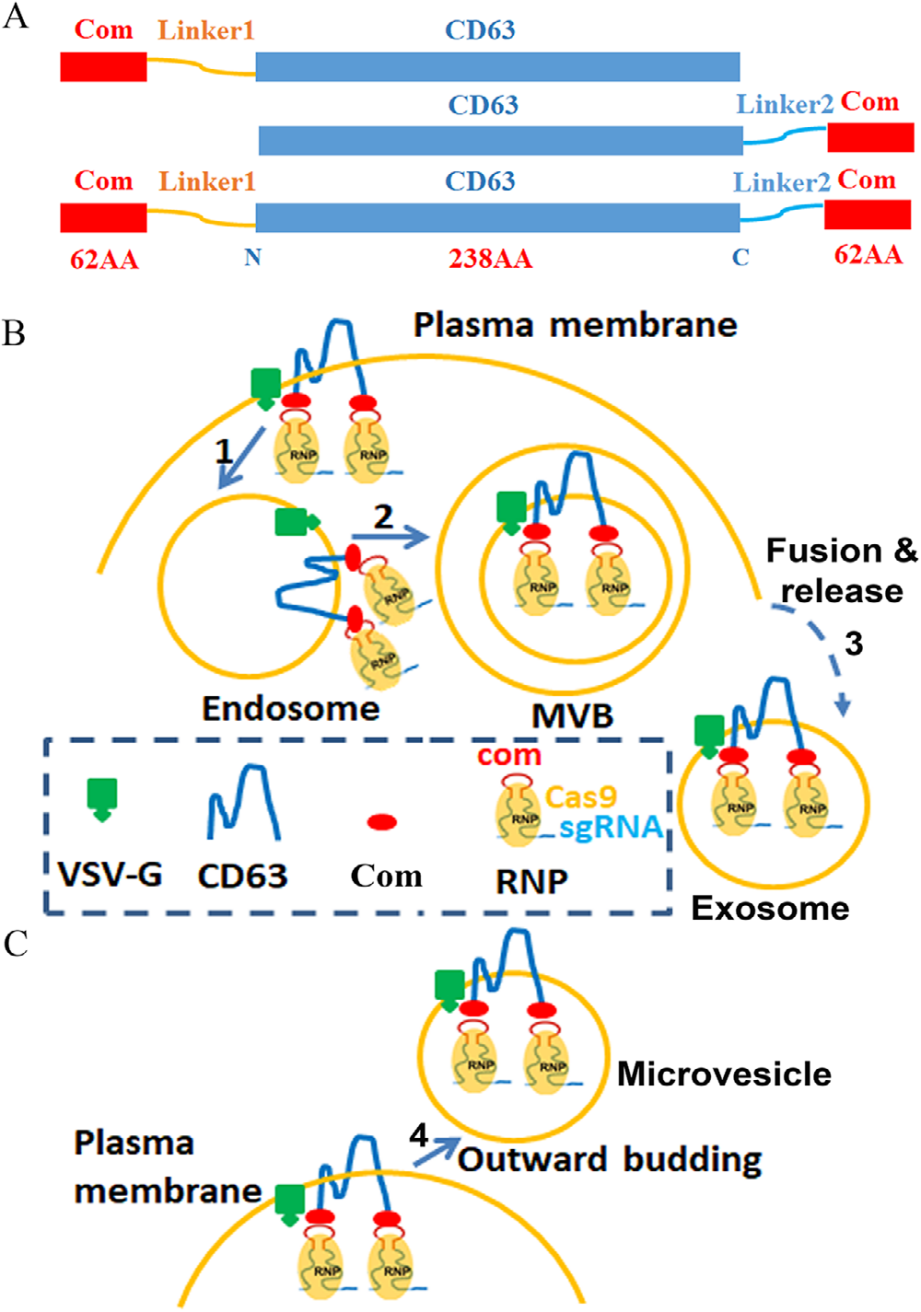
Strategy for enriching RNPs in EVs. [A. Com and CD63 fusion proteins. Com was fused to the N‐terminus, C‐terminus or both termini of CD63 with linker peptide in between. The sequence for linker 1 and linker 2 were ‘GGHNSGGGGGQSPGPAA’ and ‘SGGGGSMASNFTQFVLVDNGGTGDV’ respectively. B. Cartoon showing the recruitment of Cas9 or ABE RNPs into exosomes. RNPs associate with Com‐CD63‐Com on the plasma membrane through com/Com interaction and then enter the endosome system via endocytosis (step 1). The early endosomes become multivesicular bodies (MVB) following the inward budding of the outer endosomal membrane (step 2). The intraluminal vesicles are released into the medium as exosomes when the membranes of MVBs fuse with the cell membrane (step 3). Here Com is drawn as a monomer but may function as a homodimer. C. Cartoon showing the recruitment of Cas9 RNPs into microvesicles (step 4)]

Previously we showed that aptamer com and aptamer‐binding protein (ABP) Com were the most efficient aptamer/ABP pair for packaging Cas9 and adenine base editor RNPs into lentiviral capsids (Lu et al., 2021; Lyu et al., 2019, 2021). We decided to test whether the specific interaction between com and Com could also enrich RNPs into EVs. CD63 is a tetraspan transmembrane protein with the N‐ and C‐termini in the cytoplasm. We chose to fuse Com to the N‐terminus, the C‐terminus, or both termini of CD63 (Figure 1A), so that Com faces the cytoplasmic side of the plasma membrane. We hypothesize that during exosome generation, Cas9 or ABE RNPs will be enriched in exosomes via interactions between CD63‐Com, com‐sgRNA and Cas9 (Figure 1B). Alternatively, RNPs can also be enriched in microvesicles via the outward budding and fission of membrane vesicles from the cell surface (Figure 1C). In this study, we name the vesicle structures EVs regardless of the mechanisms of vesicle generation.

**FIGURE 2:**
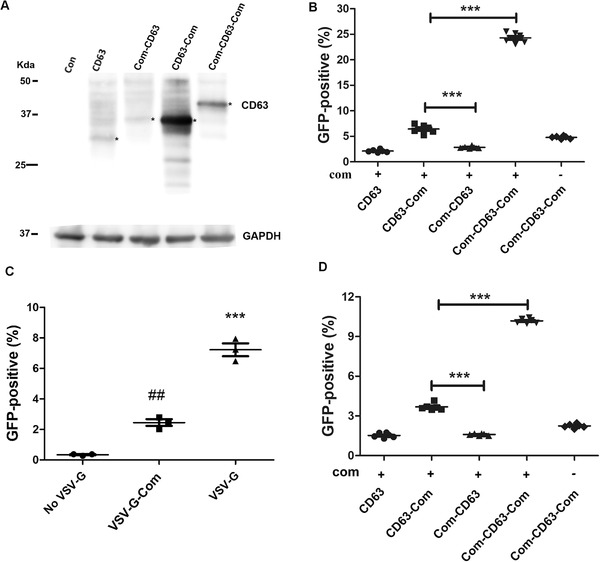
ABP and aptamer dependent enriching Cas9 RNPs into EVs. [A. Expression of Com and CD63 fusion proteins. Plasmid DNA for CD63, CD63‐Com, Com‐CD63 and Com‐CD63‐Com expression were transfected into HEK293T cells and the expression of CD63 or CD63‐fusion proteins were detected by anti‐CD63 antibody. GAPDH was detected for loading control. B. Flow cytometry detection of gene editing activities. A total of 2.5 × 10^4^ *HBB‐IL2RG* GFP reporter cells were treated with RNP‐enriched EVs secreted by 0.6 million cells in 48 h. The RNPs were *IL2RG*‐targeting SaCas9 RNPs (n = 6). ***, *P* < 0.0001 between the indicated conditions (Tukey posttests following ANOVA). C. VSV‐G was necessary for gene editing activity of the EV‐delivered RNPs. *IL2RG*‐targeting SaCas9 RNPs were packaged into EVs with or without VSV‐G protein. The RNP‐enriched EVs were added to *HBB‐IL2RG* GFP reporter cells (1/3 dosage of B) to examine GFP‐positive cells by flow cytometry (n = 3). ##, *P* < 0.01 compared with the group without VSV‐G; ***, *P* < 0.0001 when compared with all other conditions (Tukey posttests following ANOVA). D. Detection of gene editing activities of EV‐delivered SpCas9 RNPs by flow cytometry. *HBB‐IL2RG* GFP reporter cells (2.5 × 10^4^) were treated with RNP‐enriched EVs secreted by 0.6 million cells in 48 h. The RNPs were *IL2RG*‐targeting SpCas9 RNPs. ***, *P* < 0.0001 between the indicated conditions (n = 3, Tukey posttests following ANOVA)]

We first examined whether the fusion of Com affected CD63 expression, and found that fusing Com at either end did not impair CD63 expression. Indeed, fusing Com at the C‐terminus greatly increased the expression of CD63 (Figure 2A). We then tested whether Com fusion to CD63 enabled packaging of SaCas9 RNPs into EVs. Previous work found that in the recipient cells, escaping of the EVs from the endosome system is limited (Hung & Leonard, 2016), we thus over‐expressed VSV‐G in the EV producing cells so that the EVs have VSV‐G protein to facilitate their escape from the endosome system in recipient cells. Com‐CD63, CD63‐Com or Com‐CD63‐Com was co‐expressed with VSV‐G, SaCas9 and com‐modified *IL2RG*‐targeting sgRNA (Lyu et al., 2019) in HEK293T cells. The EVs were collected from the supernatant of the transfected cells and concentrated by ultracentrifugation. The resuspended EVs were added to the medium of our *HBB‐IL2RG* GFP reporter cells described previously (Javidi‐Parsijani et al., 2017). These cells expressed no EGFP due to the disruption of the EGFP reading frame by the insertion of the 119 nt *HBB* sickle mutation and *IL2RG* target sequences right after the start codon of EGFP coding sequence. INDELs formed after gene editing may restore the EGFP reading frame, resulting in EGFP expression.

Flow cytometry analyses found that without aptamer‐binding protein Com or aptamer com, few GFP‐positive cells could be observed, which could be the results of random packaging of RNPs into the EVs. When the sgRNA was modified with aptamer com, Com‐CD63‐Com generated the most GFP‐positive reporter cells, followed by CD63‐Com and Com‐CD63 (Figure 2B, see Figure S1 for representative flow cytometry data). CD63‐Com had stronger expression level than Com‐CD63, and the EV delivered RNPs also generated more GFP‐positive cells than did Com‐CD63's. The expression of Com‐CD63‐Com was weaker than that of CD63‐Com, but its EV‐associated RNPs generated significantly more GFP‐positive cells. The data showed that Com at both termini had synergistic effects on recruiting RNPs, and that both the ABP (Com) and the aptamer (com) were needed for generating EV associated RNPs with high gene editing activity.

Com‐CD63‐Com was used in further experiments since it generated EV‐associated RNPs with the highest gene editing activities. We tested different ratios of Com‐CD63‐Com and Cas9 RNP expressing plasmid DNA and found that the best activities were obtained when they were at a mass ratio of 1:4 (Figure S2).

VSV‐G was co‐expressed in the EV production cells to help the EVs escape from the endosome system in recipient cells. To check whether VSV‐G was necessary for functional delivery of the EV‐associated RNPs, we generated EV‐associated RNPs in the absence of VSV‐G. These RNPs only generated background levels of GFP‐positive reporter cells (Figure 2C), demonstrating the importance of VSV‐G. We tested whether fusing Com to the C‐terminus of VSV‐G could further increase gene editing activity, and found that this fusion greatly decreased gene editing activity of the EV‐associated RNPs. Two possibilities might underlie this observation: (1) fusing Com to the C‐terminus of VSV‐G decreased the expression of VSV‐G by over 50% (Figure S3); (2) doing so might interfere with VSV‐G's fusogenic activity.

The data showed that RNPs can be enriched in and functionally delivered by EVs, and that aptamer com, ABP Com and VSV‐G protein were all necessary for functional delivery of RNPs by EVs.

We then tested whether SpCas9 RNPs can be packaged and delivered by EVs. We recently found that replacing the ST2 loop with com aptamer best preserved the functions of SpCas9 sgRNAs and enabled efficient delivery of SpCas9 RNPs by lentivirus‐like particles (Lu et al., 2021). We tested whether similarly modified sgRNA could package SpCas9 RNPs into EVs. We co‐expressed various Com‐CD63 fusion proteins, VSV‐G, SpCas9 and ST2‐com modified *IL2RG*‐targeting sgRNA in HEK293T cells. The resultant EVs were applied to our *HBB‐IL2RG* GFP reporter cells. We found that similar to packaging SaCas9 RNPs, SpCas9 RNPs were also most efficiently packaged by Com‐CD63‐Com and both the ABP and aptamer were needed for best gene editing activities (Figure 2D). Thus the ABP/aptamer interaction can also be used to package SpCas9 RNPs into EVs.

Recently we reported that Com/com interaction can also package adenine base editor (ABE) RNPs into lentivirus‐like particles for efficient and safe base editing (Lyu et al., 2021). We wondered whether EVs can also be used to enrich and deliver ABE RNPs. We tested using EVs to package and deliver ABE RNPs targeting ABE site 5 (GRCh38.p13, chromosome 20, 32752960–32752979) (Gaudelli et al., 2017), qPCR showed that EV‐delivered site 5 ABE RNPs generated 24.3 ± 0.7 (n = 3) fold base edited products compared with EV‐delivered non‐targeting ABE RNPs. The data show that EVs can also enrich and deliver ABE RNPs.

Several studies observed that nuclear export of sgRNAs driven by U6 promoter was inefficient (Gee et al., 2020; Montagna et al., 2018). We tried to supplement RNA polymerase II promoter‐driven sgRNA (Supplementary Figure S4A), that were flanked by the Hammerhead (HH) ribozyme (Prody et al., 1986) and hepatitis delta virus (HDV) ribozyme (Nakano et al., 2000). This design has been shown to generate mature sgRNA after ribozyme cleavage (Yoshioka et al., 2015). We found that doing so only slightly increased the gene editing activities of the EV‐delivered RNPs (Figure S4B). It suggested that in our system sgRNA nuclear export was not a limiting factor, and possible explanations include that our sgRNA has com modification and that we have an active enrichment mechanism to recruit sgRNAs into membrane vesicles.

### 3.2 Com/com interaction enriched RNPs into EVs

**FIGURE 3:**
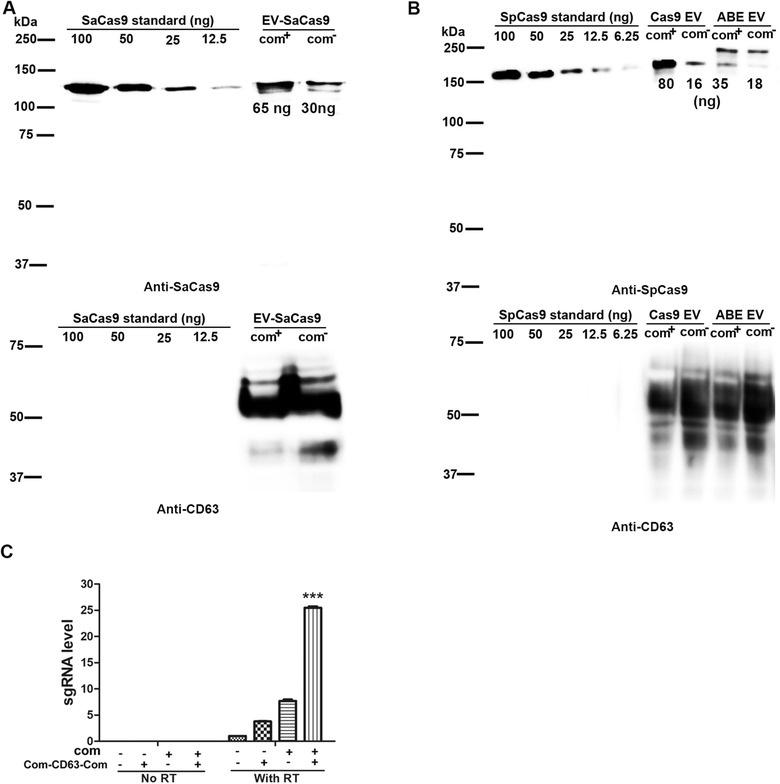
Com and com interaction enriched Cas9 RNPs into EVs. [A. Enriching SaCas9 RNPs in EVs. Com‐CD63‐Com, SaCas9 and *IL2RG* sgRNA with and without com modification were co‐expressed in 5 × 10^6^ HEK293T cells, EVs were collected for 48 h. One fifth EVs were analyzed in Western blotting. Numbers under protein bands were estimated protein mass (ng) based on protein standards. B. Enriching SpCas9 and ABE RNPs into EVs. *IL2RG* and Site 5 were targeted for SpCas9 and ABE respectively. Experimental conditions were similar as in A. C. Com and com dependent enrichment of sgRNA into EVs. SaCas9 and *IL2RG*‐targeting sgRNA were packaged in EVs (n = 3). sgRNA was unmodified (com−) or with a com replacing the Tetra loop (com+). In EVs without Com‐CD63‐Com, CD63 was overexpressed instead. RNAs were extracted from EVs and the sgRNA was detected by RT‐qPCR using primers Scid‐g2F and sgRNA‐R3 (See Table S2). ***, *P* < 0.0001 when compared with all other conditions (Tukey posttests following ANOVA)]

We examined whether Com/com interaction enriched RNPs into EVs by Western blotting. We prepared EVs loaded with RNPs containing sgRNAs with and without com aptamer and detected SaCas9, SpCas9 and ABE content in the respective EVs. We detected 2–5 fold Cas9 or ABE protein in RNPs with com‐modified sgRNAs (com^+^) compared with RNPs with unmodified sgRNAs (com^−^), although the amount of Com‐CD63‐Com protein was comparable (Figure 3A, B). Consistent with these observations, the presence of Com and com packaged the most sgRNA in EVs (Figure 3C). Although the presence of non‐functional RNPs associating with the membranous structures non‐specifically (Lu et al., 2021) underestimated the enrichment effects of Com/com interaction, the data showed that Com/com interactions enriched RNPs into EVs.

Using the Pierce BCA Protein Assay Kit, we found that 1 million transfected cells secreted about 625 ng total EV proteins in 48 h. Among which, 65 ng SaCas9, 80 ng SpCas9 and 35 ng ABE proteins were detected based on Western blotting of Cas9 proteins of known concentrations (Figure 3A, B).

### 3.3 EV delivered RNPs achieved efficient gene editing on multiple targets in different cells

We further confirmed genome editing activities of the EV delivered RNPs targeting various targets in different cells by NGS. As shown in Table 1 and Figure S5, we observed INDELs in different lentiviral integrated targets in GFP‐reporter cells, in HEK293T cells where the EVs were generated, and in MDA‐MB‐231 cells that are different from the EV source cells and are hard to transfect. We also observed A to G changes on site 5 in different cells treated with EV‐delivered ABE RNPs (Table 1 and Figure S5D).

**TABLE 1:** NGS analyses of INDEL rates and base editing rates of EV‐delivered RNPs

		SpCas9				
	SaCas9 IL2RG	DMD	GAPDH	P53	Site 5	ABE Site 5
GFP‐reporter cells	48.2%^a^	51.4%^b^	ND	ND	ND	ND
HEK293T	ND	ND	15.7%^c^	16.1%^d^	35.5%^d^	39.7%^c^
MDA‐MB‐231	ND	ND		14.4%^d^	33.8%^d^	24.2%^c^

For all assays, EVs were added to 2.5 × 10^4^ cells.

^a^EVs produced by 0.6 million cells in 48 h were added to *HBB‐IL2RG* GFP‐reporter cells.

^b^EVs produced by 2.5 million cells were added to *DMD* GFP‐reporter cells.

^c^EVs produced by 5 million cells were added to the respective cells.

^d^EVs produced by 20 million cells were added to the respective cells.

Based on our estimation of production rates for EV‐associated RNPs (Figure 3), the amounts of EV‐delivered RNPs used in these experiments were between 0.13 and 6.4 μg for 10^5^ cells. The highest dosage was lower than that used in a typical electroporation experiment (10 μg).

### 3.4 Characterization of EVs with RNP enrichment

We examined whether over‐expressing Com‐CD63‐Com affected EV biogenesis by isolating EVs from cells with and without Com‐CD63‐Com overexpression, and examining protein expression by Western blotting. We found that the expression of exosome marker proteins CD9 and RAB5B was not decreased from EVs isolated from cells with Com‐CD63‐Com overexpression (Figure S6A), suggesting that Com‐CD63‐Com overexpression did not decrease overall EV biogenesis. In addition, the overexpression of Cas9 RNPs did not impair EV biogenesis either. Com‐CD63‐Com overexpression greatly increased the detection of CD63‐reactive antigens in EVs. The observed CD63 size was much larger than expected, and the bands appeared diffused. Both observations were the results of heterogeneous glycosylation of CD63. For the same reason, the size difference between endogenous CD63 and the overexpressed Com‐CD63‐Com were not obvious. Compared with cellular Com‐CD63‐Com (Figure 2A), Com‐CD63‐Com in EVs apparently appeared larger and more heterogeneous in size. It seems that CD63 proteins in EVs had a high degree of glycosylation. As expected, VSV‐G and Cas9 were only observed in EVs from cells overexpressing the respective protein. GRP94, a protein not involved in the endosome pathway, was not observed in the EV preparations, suggesting minimal cellular protein contamination.

Transmission electron microscopy was performed to examine the morphology of the EVs isolated from cells without CD63 overexpression, with CD63 or Com‐CD63‐Com overexpression (all cells overexpressed VSV‐G and Cas9 RNPs). No difference was observed in morphology of the EVs isolated from these cells (Figure S6B). We performed nanoparticle tracking analysis (Nanosight) to examine the particle number and size of the EVs isolated from the cells, and found that overexpressing Com‐CD63‐Com and Cas9 RNPs slightly decreased the total number of EV particles generated (Figure S6C). In addition, overexpressing Com‐CD63‐Com and Cas9 RNPs changed the EV size distribution, with a decrease of EVs of 100 nm in diameter and an increase of EVs of 200 nm in diameter (Figure S6D). The right shift of size distribution explained why CD9 and RAB5B expression in EVs was not decreased although the total EV number was decreased with Com‐CD63‐Com overexpression.

### 3.5 Short half‐life of Cas9 RNPs delivered by EVs

In order to examine the fate of EV delivered RNPs in human cells, we added EV delivered, *DMD* exon 53‐targeting SpCas9 RNPs to HEK293T cultures, and collected the cells at different time after EV addition. Western blotting found that 6 h after delivery, the SpCas9 protein level was the highest and thereafter SpCas9 protein level decreased quickly (Figure S7A). Densitometry analysis revealed that 18 h post‐delivery, Cas9 level was only 10% of that of 6 h post‐delivery (Figure S7B). The half‐life of EV delivered SpCas9 protein in human cells was estimated to be 3 h.

We did similar experiments on EV delivered, *IL2RG*‐targeting SaCas9 RNPs. In this experiment, the relative low level of SaCas9 protein delivered by the EVs reached the limitation of the SaCas9 antibody sensitivity and specificity, but we could still observe a band with the expected size that was only observed in treated cells (Figure S7C). This SaCas9‐specific band also showed similar quick degradation. Thus Cas9 RNPs delivered by EVs had very short half‐lives. This short half‐life ensures the specificity and safety of EV delivered RNPs.

### 3.6 Single preparation of RNP‐enriched EVs for multiplex gene editing

Since each CD63 molecule is fused to two Com molecules, and each EV may have more than one Com‐CD63‐Com molecule, we reasoned that EVs could be ideal for delivering RNPs for simultaneously targeting multiple loci.

**FIGURE 4:**
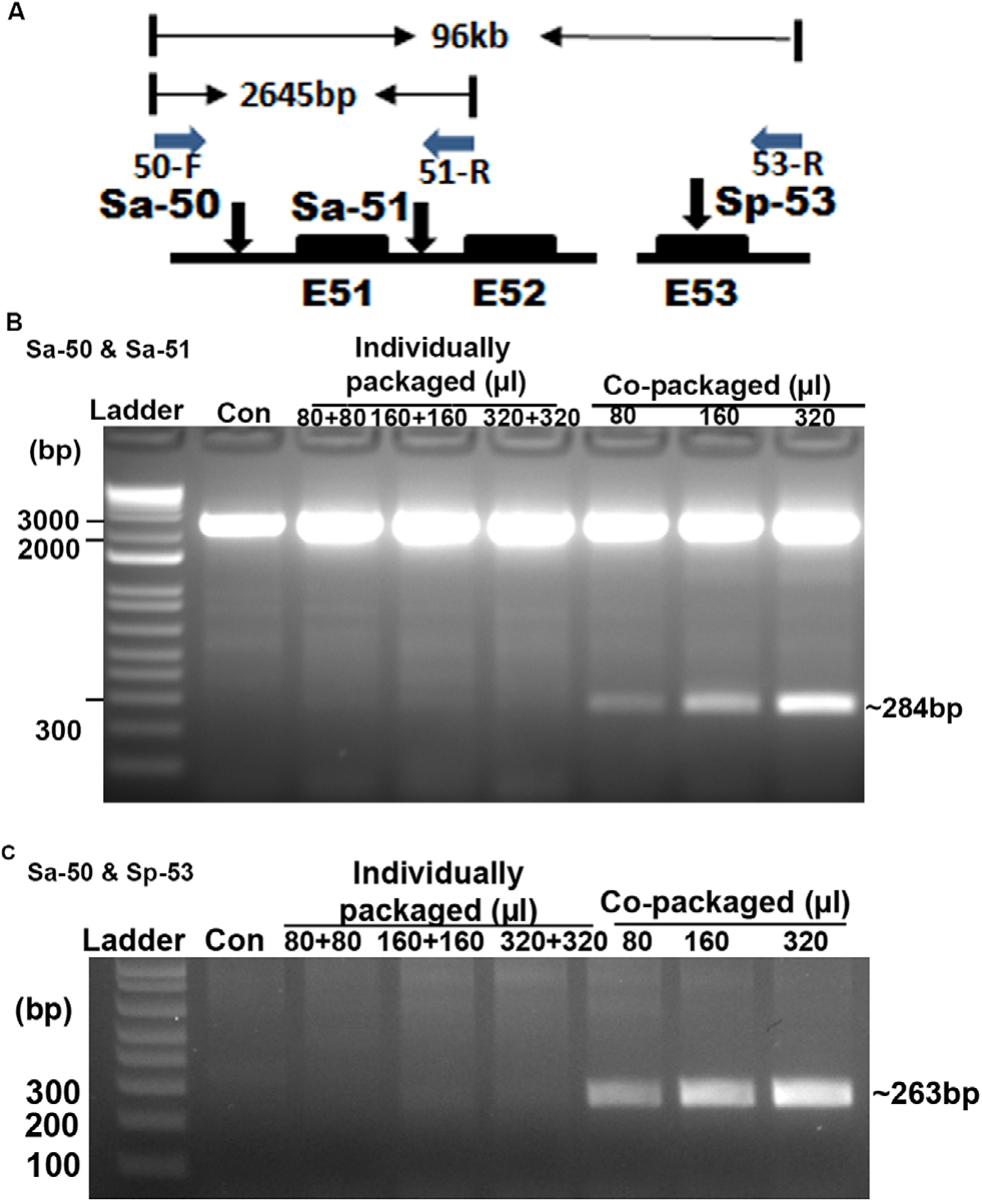
Co‐packaging of RNPs for efficient multiplex genome editing and long‐range genomic deletion. [A. Diagram showing the strategy to detect co‐targeting of both loci. The three sgRNAs, Sa‐50, Sa‐51 and Sp‐53, target DMD intron 50, intron 51 and exon 53 respectively. The primers, 50‐F, 51‐R and 53‐R used for PCR detection of deletions are also shown. The solid boxes indicate hDMD exons 51 to 53. Distances between primers before sequence removal are listed. B. Co‐packaged SaCas9 RNPs targeting different loci were more efficient in multiplex genome editing. C. SaCas9 RNPs and SpCas9 RNPs could be co‐packaged in EVs for efficient multiplex genome editing]

We first tested using two SaCas9 RNPs, targeting *DMD* intron 50 (Sa‐50) and intron 51 (Sa‐51) respectively, to remove the 2361 bp between the two target sites (Figure 4A). We prepared EVs loaded with RNPs targeting *DMD* intron 50, RNPs targeting *DMD* intron 51, and RNPs targeting both introns. EVs loaded with RNPs targeting both introns were prepared in a single EV preparation simply by using half of each target plasmid DNA. The two individually packaged RNPs were used together to compare with the co‐packaged RNPs for exon 51 removal. All RNP‐loaded EVs were prepared in parallel and similarly concentrated. PCR was used to detect exon 51 removal: a 2645 bp would be amplified with primers DMD50‐F and DMD51‐R2 without exon 51 removal (Figure 4A), otherwise a 284 bp product would be amplified. We could only observe a 284 bp amplicon in cells treated with the co‐packaged RNPs, but not in cells treated with the two individually packaged RNPs (Figure 4B). DNA sequencing confirmed that the ∼284 bp amplicon was the results of deleting the sequences between the two sgRNAs (Figure S8). This experiment showed that one single preparation can produce EVs loaded with RNPs targeting more than one locus, and doing so is more efficient for multiplex gene targeting.

We also tested packaging SaCas9 and SpCas9 RNPs in one single preparation. In this case, *DMD* intron 50‐targeting SaCas9 RNPs and *DMD* exon 53‐targeting SpCas9 RNPs were individually packaged or co‐packaged into EVs. The two individually packaged RNPs were used together to compare with the co‐packaged RNPs for removing the sequences between the two target sites 96 kb away (Figure 4A). PCR showed that the expected 263 bp DNA amplicon, indicating the deletion of the 96 kb and confirmed by DNA sequencing (Figure S8), were only observed in cells treated with the co‐packaged RNPs but not in cells treated with the two individually packaged RNPs (Figure 4C). Thus RNPs of Cas9 from different species (e.g., SaCas9 and SpCas9) can also be co‐packaged into EVs in single preparation for efficient multiplex gene editing.

### 3.7 In vivo activity of EV delivered RNPs

**FIGURE 5:**
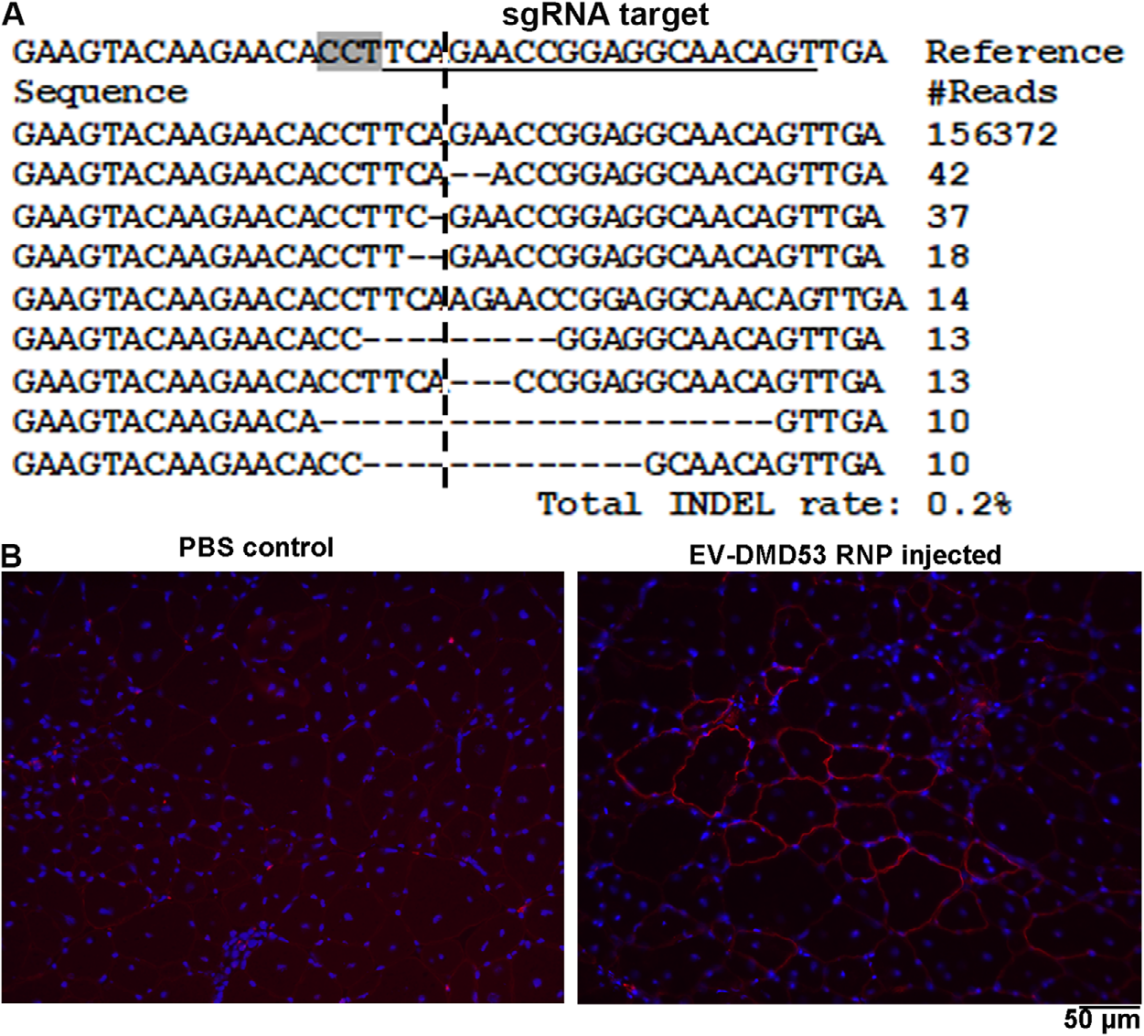
EV for in vivo Cas9 RNP delivery. [A. NGS analysis of INDELs in TA muscles after EV delivered RNP treatment. The TA muscle was injected with EVs produced by 40 million cells in 48 h. Sequences observed in one of the treated legs were listed. The target sequence for sgRNA is underlined and the PAM region is highlighted. The predicted cleavage site is indicted by a vertical dashed line. B. Immunostaining of dystrophin in control and DMD Cas9 RNP injected TA muscles. The TA muscle was injected with EVs produced by 2 million cells in 48 h]

We further examined the in vivo activity of EV delivered *DMD* exon 53‐targeting RNPs. RNP‐loaded EVs produced by 40 million cells in 48 h were concentrated and injected into each TA muscle of del52hDMD/*mdx* mice (Veltrop et al., 2018). One week later the mice were sacrificed and the TA muscle were collected to examine for target gene editing. We isolated gDNA from the TA muscle and amplified the target DNA region for NGS analysis. We observed up to 0.2% INDEL rates (316 of 157982 reads) in the RNP injected muscle and 0% INDEL rate in the PBS injected muscle (0 of 51302 reads, *P* < 0.0001 by Chi‐square tests). The INDELs were all round the predicted cleavage site (Figure 5A).

We performed immunostaining to examine the expression of dystrophin in injected TA muscle. As we described recently (Lyu et al., 2020), these mice have low background dystrophin expression in skeletal muscle due to spontaneous exon 53 skipping which restores the dystrophin reading frame. In RNP injected TA muscle, we observed areas with stronger dystrophin expression that were not observed in the PBS injected muscles (Figure 5B).

## 4 DISCUSSION

EVs have been used to deliver Cas9 RNPs for genome editing (Campbell et al., 2019; Chen et al., 2019; Gee et al., 2020; Kim et al., 2017; Lainscek et al., 2018; Montagna et al., 2018; Ye et al., 2020). However, enriching RNPs into EVs is yet to be achieved. One group enriched Cas9 RNPs into EVs by fusing GFP to exosome enriched protein CD63, and fusing single chain GFP‐binding antibody to Cas9 (Ye et al., 2020). However, they only observed moderate genome editing efficiency. Another group fused FKBP12 with lentiviral Gag protein, and fused FRB with SpCas9 protein (Gee et al., 2020). In the presence of rapamycin analog AP21967, FKBP12 and FRB interaction is induced, which brings SpCas9 protein to Gag. The resultant ‘extracellular nanovesicles’ are more likely virus‐like particles rather than typical EVs without viral capsids. Thus current methods using EVs to deliver Cas9 RNPs either do not have mechanisms to actively enrich RNPs into EVs, or have mechanisms that are non‐specific or inefficient.

Here we develop a method to actively enrich Cas9 and ABE RNPs into EVs. We used the specific interaction between aptamer com (inserted in sgRNA) and aptamer‐binding protein Com (fused to CD63). The specific com/Com interaction associates RNPs to the N‐ and C‐termini of CD63, which is in the cytoplasm of the cells and the lumen of exosomes. Since CD63 is abundant on exosomes (Escola et al., 1998; Kobayashi et al., 2000), our method is expected to specifically enrich Cas9 and ABE RNPs into EVs. Indeed, with our enriching mechanism, up to 10 times more gene editing activity was observed compared with EVs without RNP enriching mechanism (e.g., without aptamer com or aptamer‐binding protein Com). Since the Cas9 and sgRNA expressing plasmid DNA were also present in conditions without RNP enriching mechanism, the low gene editing activity observed in these conditions ruled out a major contribution of EV transferred plasmid DNA.

One interesting observation was that adding aptamer‐binding protein Com to both the N‐ and C‐termini of CD63 showed a synergistic effect on gene editing activity. There could be several explanations to this observation: (1) Com may function as a homodimer and fusing Com to both the N‐ and C‐termini of CD63 increases the chance of making a functional unit. If this is true, fusing two tandem copies of Com at each ends of CD63 may further increase gene editing activity. This will be tested in further studies. (2) A threshold of Cas9 molecules are needed to be functional and fusing Com to both the N‐ and C‐termini of CD63 increases the chance of reaching that threshold.

Another interesting observation is that VSV‐G is important for genome editing activity for the EV delivered RNPs. The most likely explanation is that VSV‐G helps the escape of the RNPs from the endosome system in recipient cells. Indeed, studies showed that miRNA and mRNA delivered by EVs without VSV‐G are non‐functional due to quick degradation in recipient cells (Hung & Leonard, 2016; Kanada et al., 2015). We added Com to the C‐terminus of VSV‐G and this manipulation also diminished activity of EV delivered RNPs. It seems that an intact and free C‐terminus is important for VSV‐G to induce endosome escape. Accordingly, when fusing MS2‐binding protein or Hemagglutinin tag to the C‐terminus of VSV‐G, the EV delivered mRNA was also not translated (Hung & Leonard, 2016). Recently it is found that connexin 43, especially the constitutively active S368A mutant can functionally deliver EV cargoes into the cytoplasm of the recipient cells (Kojima et al., 2018). It is interesting to know whether connexin 43 S368A mutant can replace VSV‐G in our system considering the possible cellular toxicity of VSV‐G protein.

Advantages of our EV mediated RNP delivery system are that RNPs targeting more than one loci and RNPs with Cas9 proteins from different species can be enriched in EVs in a single RNP preparation. We demonstrated the co‐packaging of SaCas9 and SpCas9 RNPs. It is expected that Cas9 proteins from other species may also be co‐packaged as long as the com aptamer can be inserted into their sgRNA. In addition, the co‐packaged RNPs are much more active than the combination of the individually packaged RNPs for multiplex genome editing. These features make EVs an ideal delivery tool for multiplex genome editing, which is needed in many situations, including knockout of antigens to reduce the risk of immuno‐rejection (Fischer et al., 2019; Han et al., 2019; Ren et al., 2017), eradicating HIV proviral DNA from genome (Kaminski et al., 2016), and enhancing response in cancer therapy (Stadtmauer et al., 2020).

We modified the sgRNA scaffolds the same way for EV and lentivirus‐like particle mediated RNP delivery. Whereas the RNP‐loaded EVs typically need to be concentrated for 20∼100 times before use, the RNP‐enriched lentivirus‐like particles usually generated higher gene editing activities on the same targets without concentration (Lu et al., 2021; Lyu et al., 2019, 2020, 2021). There could be several explanations to the difference. First, available data showed that the half‐life of EV‐delivered RNPs was shorter than that of virus‐like particle delivered RNPs. For SpCas9 RNPs, the half‐lives for EV and virus‐like particle delivered RNPs were 3 h (this study) and 7.3 h (Lu et al., 2021) respectively. Second, the generation of EVs could be less efficient than generating virus‐like particles. Third, the RNPs could be better protected by virus‐like particles in recipient cells due to the presence of viral capsids. And finally, the dissociation of RNPs from CD63 may be inefficient after escaping from the endosome. Optimizing Com copy number, improving RNP escape from the endosome system and optimizing EV source cells may help to improve the EV RNP delivery system.

However, EV as a delivery vehicle for RNPs may have advantages than virus‐like particles. One advantage is that EVs could be more suitable for systemic delivery than virus‐like particles, which tend to be inactivated by complement system in circulation. Another one is the capability of EVs to cross the blood brain barrier. We have demonstrated genome editing after EV mediated local delivery, but have yet to demonstrate the capability of systemic delivery in future work.

## FUNDING

This work was partially supported by DOD (W81XWH2010265 to Baisong Lu), The Bruce D. and Susan J. Meyer Charitable Fund (to Baisong Lu) and the state of North Carolina (Grant 330054 to Anthony Atala).

## Supporting information

Supporting Information

Supporting Information

Supporting Information

Supporting Information

Supporting Information

Supporting Information

Supporting Information

Supporting Information

Supporting Information

Supporting Information

Supporting Information

## Data Availability

All data that support the findings of this study are available in the paper and its Supplementary Information.
