# Supplementary figures and images for: Engineered extracellular vesicles as versatile ribonucleoprotein delivery vehicles for efficient and safe CRISPR genome editing
Source: J Extracell Vesicles. 2021 Mar 16;10(5):e12076. doi: 10.1002/jev2.12076 (PMC7962171; doi:10.1002/jev2.12076)

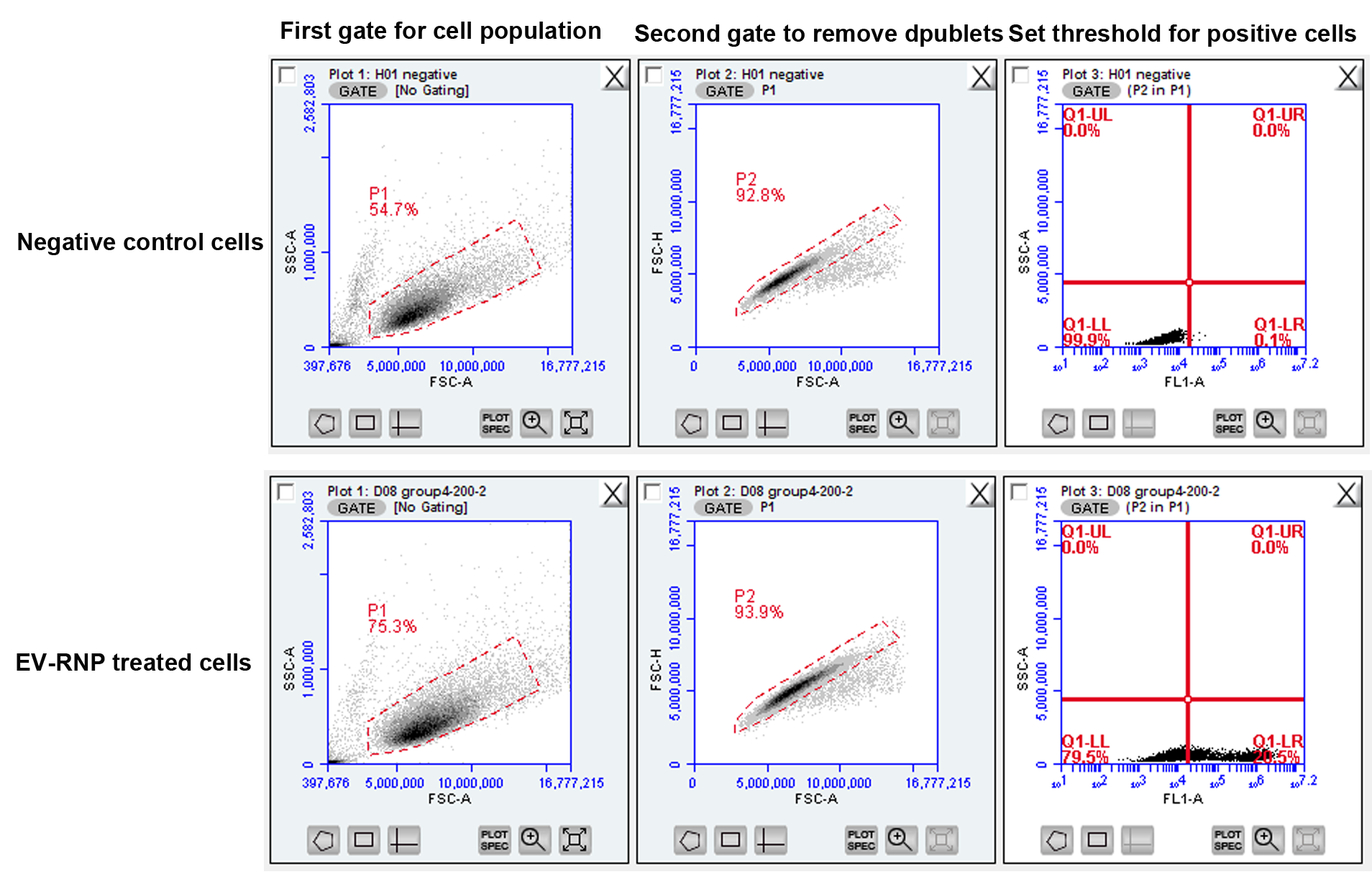

Supplement: Supplementary file 1 — Supporting Information [file JEV2-10-e12076-s009.tif]

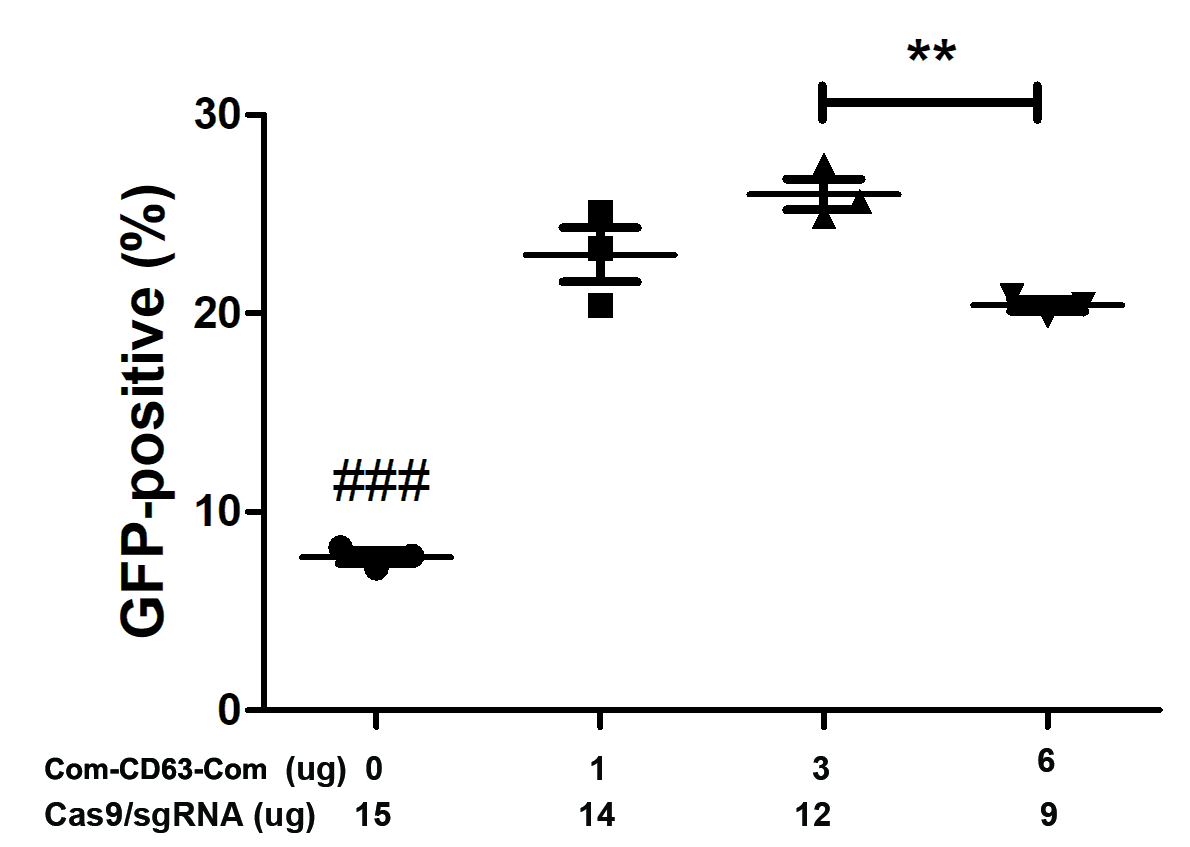

Supplement: Supplementary file 2 — Supporting Information [file JEV2-10-e12076-s007.tif]

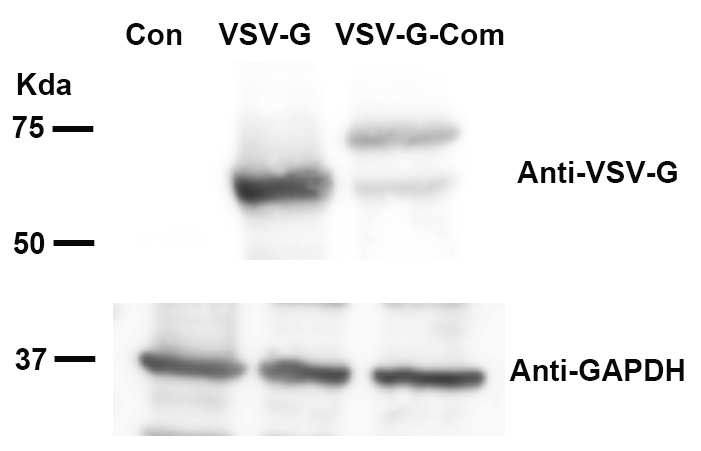

Supplement: Supplementary file 3 — Supporting Information [file JEV2-10-e12076-s003.tif]

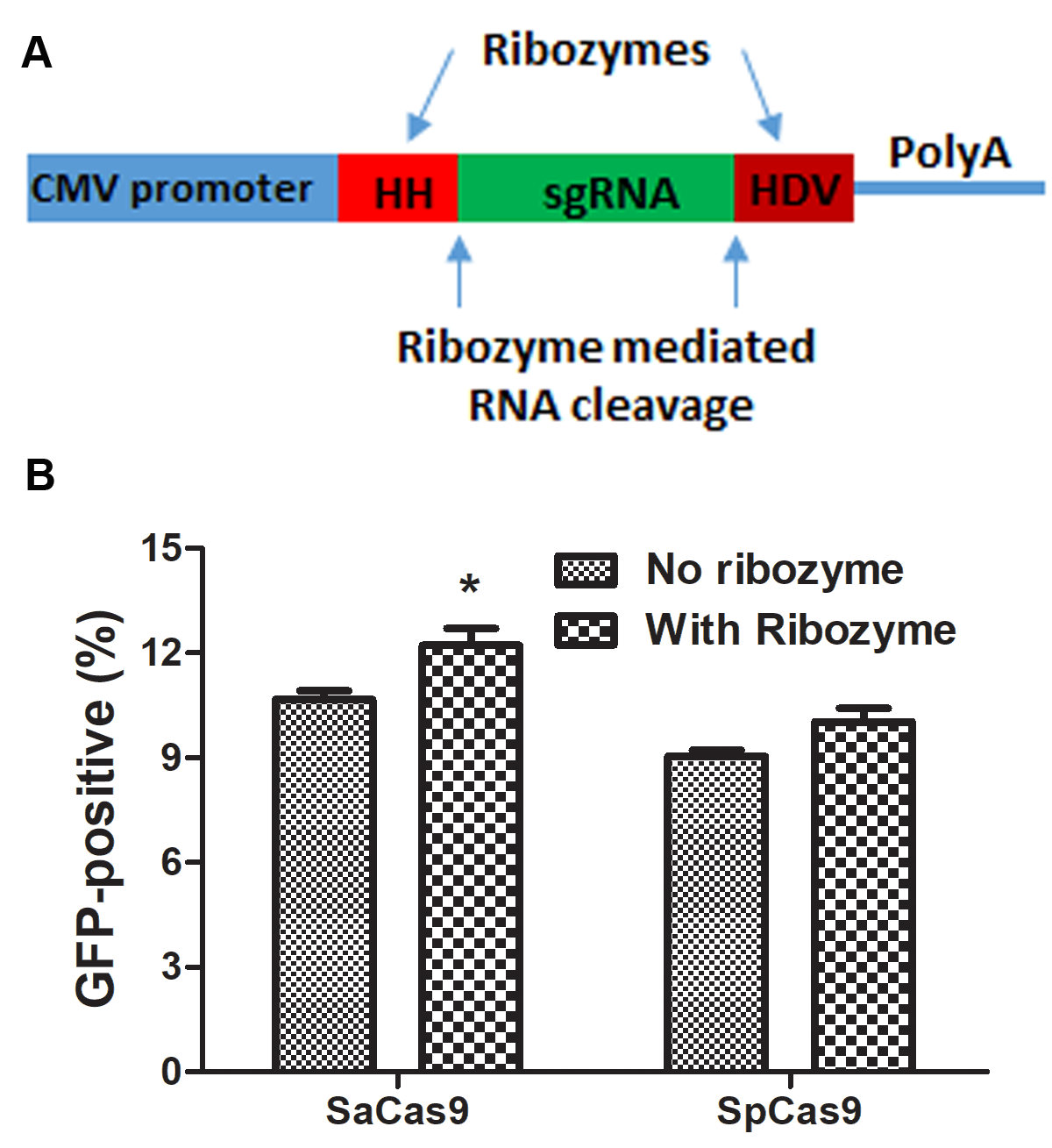

Supplement: Supplementary file 4 — Supporting Information [file JEV2-10-e12076-s011.tif]

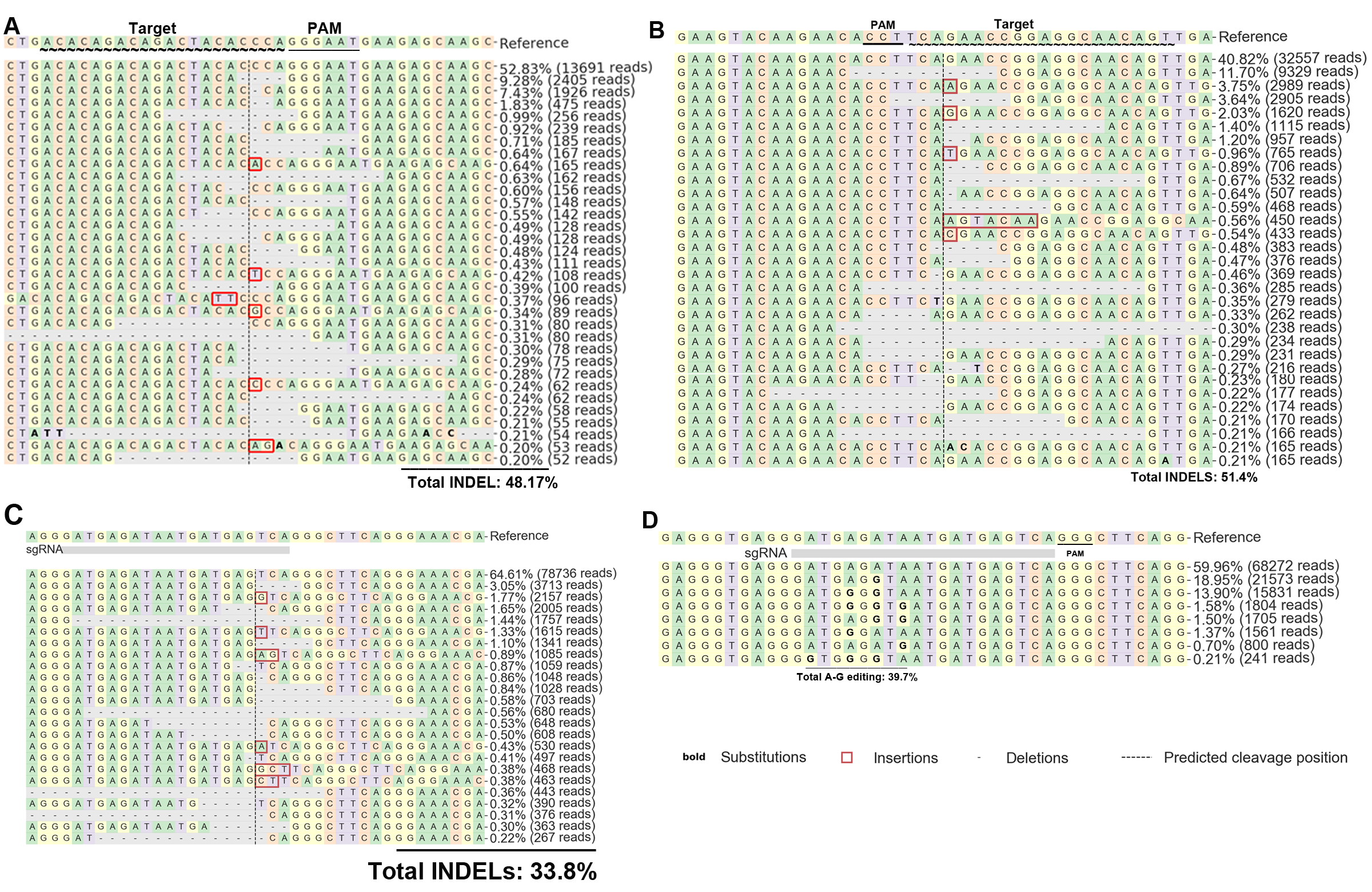

Supplement: Supplementary file 5 — Supporting Information [file JEV2-10-e12076-s002.tif]

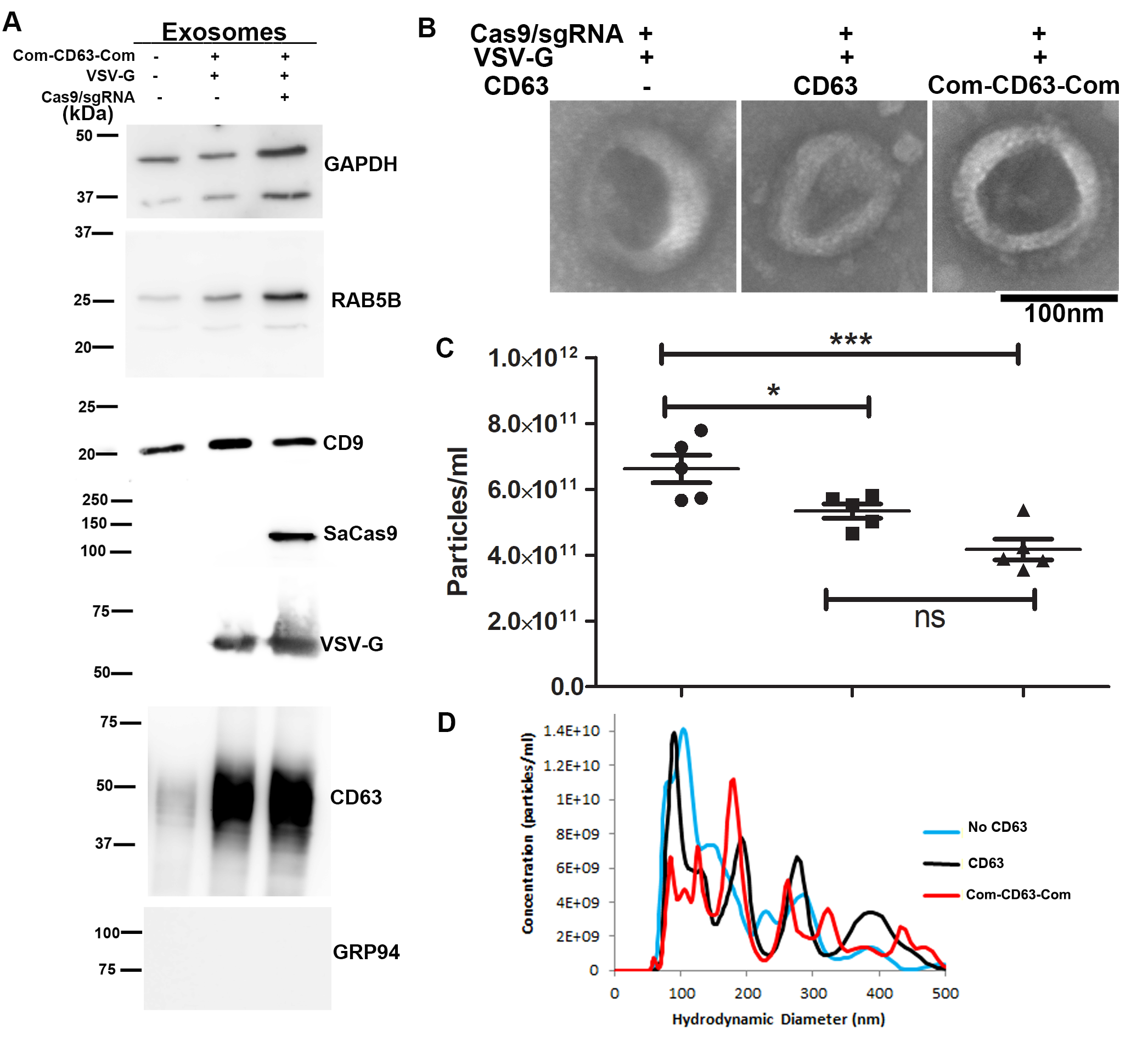

Supplement: Supplementary file 6 — Supporting Information [file JEV2-10-e12076-s006.tif]

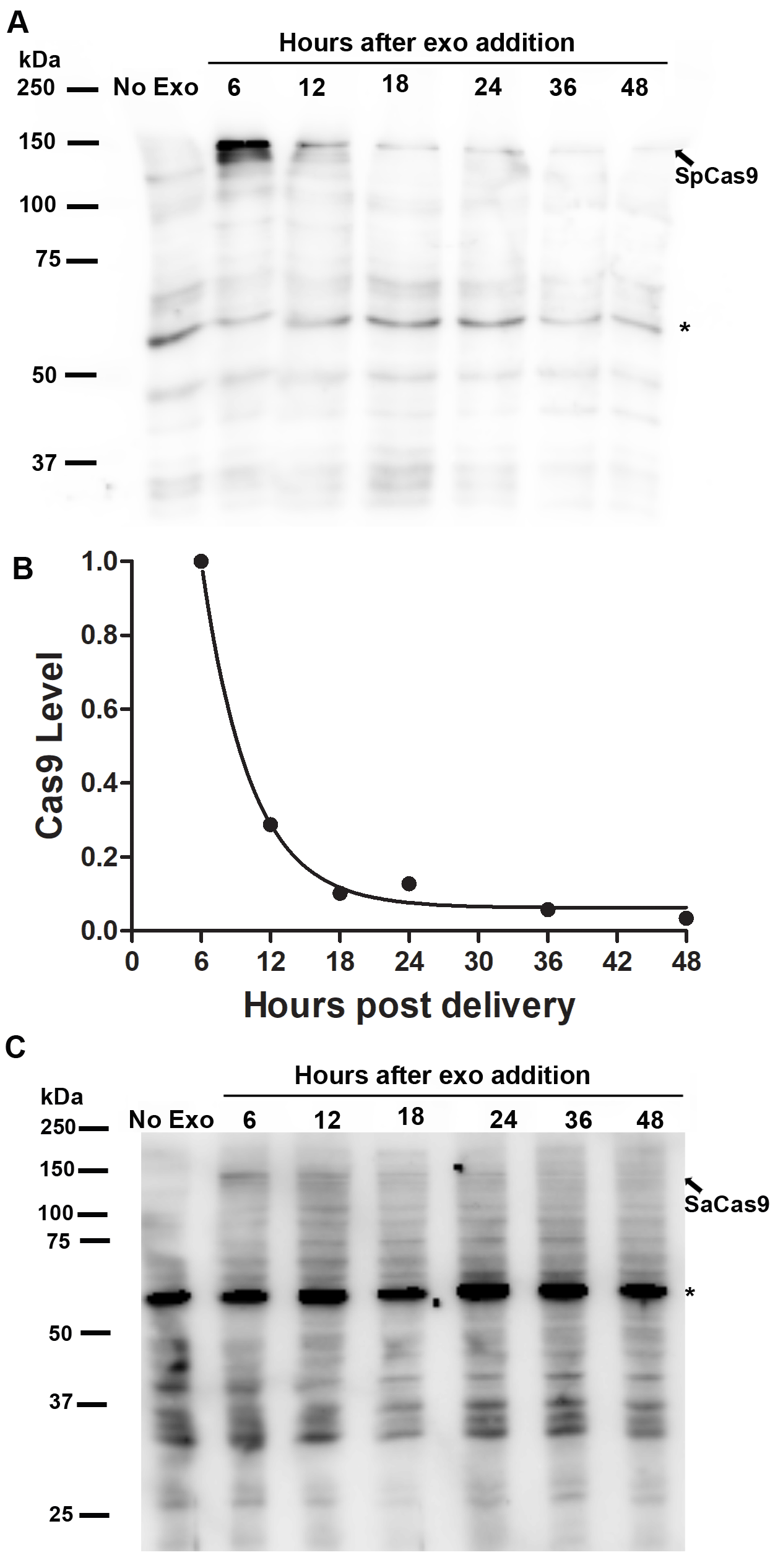

Supplement: Supplementary file 7 — Supporting Information [file JEV2-10-e12076-s005.tif]

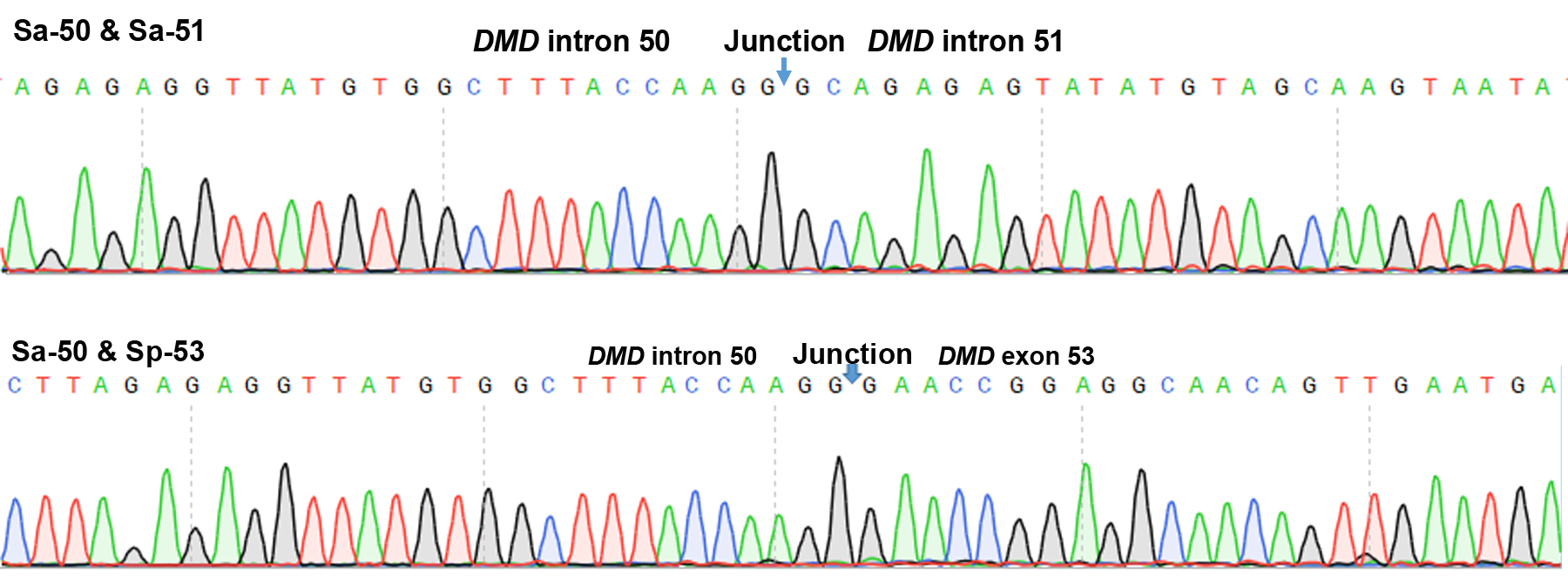

Supplement: Supplementary file 8 — Supporting Information [file JEV2-10-e12076-s004.tif]
