# Supplementary material for: Engineered extracellular vesicles as versatile ribonucleoprotein delivery vehicles for efficient and safe CRISPR genome editing
Source: J Extracell Vesicles. 2021 Mar 16;10(5):e12076. doi: 10.1002/jev2.12076 (PMC7962171; doi:10.1002/jev2.12076)
Supplement: Supplementary file 9 — Supporting Information [file JEV2-10-e12076-s010.docx]

**Supplementary Table S1. Plasmids used for the study**

| No. | Name | Purpose | Generation strategy |
| --- | --- | --- | --- |
| 1 | pMD2G-com | Mammalian expression plasmid expressing VSV-G with a COM aptamer binding protein at the C-terminus. | Use VSV-F (agatgaaccgacttgGaaagtccggtggaggtggatccat) and VSV-R (ccacaccagccaccaATCAATAACGCACGGTTTCGTCAGAATGCGT) to amplify a 300 bp fragment encoding COM (the com aptamer in COM coding DNA was disrupted) from pspAX2-D64V-NC-com-new 2. The 300 bp DNA was inserted into the BsgI site of pMD2G by Infusion reaction. |
| 2 | pMD2G-IRES-T7 | Mammalian expression plasmid expressing VSV-G and T7 RNA polymerase from one cistron linked with IRES sequence. | Use IRES-T7-F (agatgaaccgacttggaaagtaacacaacggtttccctctagc) and IRES-T7-R (ccacaccagccaccactcgagtgcttccttcacgacattca) to amplify a 291 bp band from pT7T7 (Gift from Thomas E. Wagner), which is inserted into the BsgI site of pMD2G by Infusion reaction. This plasmid is named pMD2G-IRES-partial. Then the AVrII-XhoI fragment from pT7T7 was inserted into AVrII-XhoI sites of pMD2G-IRES-partial. |
| 3 | pCOM-CD63 | Mammalian expression plasmid expressing a fusion protein COM-CD63 (COM at the N-terminus). | Use COM-CD63-F1 (cgtcagatccgctagCCACCATGAAATCAATTCGCtgtaaaaactg) and COM-CD63-R1 (CGGGCCTGGGCTCTGTCCACCTCCACCTCCGGAGTTGTGACCGCCATAACGCACGGTTTC) to amplify the COM coding region 251 bp from pspAX2-D64V-NC-com-new 2. The fragment was inserted between the NheI-KpnI sites of plasmid CD63-pEGFP C2 (Addgene # 62964) by Infusion reaction. |
| 4 | pCD63-COM | Mammalian expression plasmid expressing a fusion protein CD63-COM (COM at the C-terminus). | Use CD63-COM-F1 (ttgggggctggctgaggaa) and CD63-COM-MR (ccatggatccacctccaccggacatcacctcgtagccacttc) to amplify a 153 bp fragment from pCD63-EGFP-C2 (Addgene ID 62964). Use CD63-COM-MF (gaagtggctacgaggtgatgtccggtggaggtggatccatgg) and CD63-COM-R (Tagatccggtggatcagatctctaataacgcacggtttcgtcag) to amplify the 305 bp fragment encoding COM. Then use the two PCR fragment as the template and use CD63-COM-F1 and CD63-COM-R to amplify the 416 bp fragment, which is then inserted into BbvC1-BamH1 sites of plasmid CD63-pEGFP C2 (Addgene # 62964) by Infusion reaction. |
| 5 | pCOM-CD63-COM | Mammalian expression plasmid expressing a fusion protein COM-CD63-COM (COM at both ends of CD63). | The NheI-BbsI fragment of pCOM-CD63 was inserted into the NheI-BbsI sites of pCD63-COM. |
| 6 | pCMV-ribo-sgRNA-sp vector | A vector expressing RNA polymerase II promoter controlled sgRNA for SpCas9, with rybozymes for self-cleavage. | pCMV-PE2 was cut with NotI and PmeI to remove the PE2 cDNA after the CMV promoter. A synthesized DNA “gcggccgcAGCAGTGGCGCCCGAACAGGGACTTGAAAGCGAAAGGGAAACCAGAGGAGCTCTCTCGACGCAGGACTCGGCTTGCTGAAGCGCGCACGGCAAGAGGCGAGGGGAGGCGACTGGTGAGTACGCCAAAAATTTTGACTAGCGGAGGCTAGAAGGAGAGAGATGGGTGCGAGAGCGTCAGTATTAAGCGGGGGAGAATTAGATCGATGGacAAGCTTAGGAGCTTTGTTCCTTGGGTTCTTGGGAGCAGCAGGAAGCACTATGGGCGCACGGTCAATGACGCTGACGGTACAGGCCAGACAATTATTGTCTGGTATAGTGCAGCAGCAGAACAATTTGCTGAGGGCTATTGAGGCGCAACAGCATCTGTTGCAACTCACAGTCTGGGGCATCAAGCAGCTCCAGGCAAGAATCCTGGCTGTGGAAAGATACCTAAAGGATCAACAGCTCCTacccggagagacgggatcccgtctctGTTTGAGAGCTAGAAATAGCAAGTTCAAATAAGGCTAGTCCGTTATCAACTTGGCTGAATGCCTGCGAGCATCCCACCCAAGTGGCACCGAGTCGGTGCTTTTGGCCGGCATGGTCCCAGCCTCCTCGCTGGCGCCGGCTGGGCAACATGCTTCGGCATGGCGAATGGGACgtttaaac” was cut with NotI and PmeI and was inserted into the vector DNA prepared as described above by T4 DNA ligase. The synthesized DNA include HIV ψ signal, HIV Rev responsive element, com-modified sgRNA for spCas9, and HDV ribozyme sequence. |
| 7 | pCMV-ribo-sgRNA-Sa vector | A vector expressing RNA polymerase II promoter controlled sgRNA for SaCas9, with rybozymes for self-cleavage. | pCMV-ribo-sgRNA-sp vector was cut with BamH1 and pmeI to remove the sgRNA scaffold for SpCas9. Then , pX601-Tetra-com-vector was used as the template to amplify the 136 bp with primer Sa-ribo-F  (cggagagacgggatcccgtctctgtttaagtactctgCTGAATG) and Sa-Ribo-MR  (GGACCATGCCGGCCAAAAtctcgccaacaagttgacg); pCMV-ribo-sgRNA-sp vector was used as the template to amplify the 106 bp DNA with primers  Sa-Ribo-MF  (cgtcaacttgttggcgagaTTTTGGCCGGCATGGTCC) and Sa-Ribo-R  (ctgatcagcgggtttGTCCCATTCGCCATGCCGA); The two PCR products were used as the template, and Sa-ribo-F and Sa-Ribo-R were used as primers to do overlap PCR to obtain the 205 bp fragment, which include HIV ψ signal, HIV Rev responsive element, com-modified sgRNA for SaCas9, and HDV ribozyme sequence. The 205 bp fragment was inserted into the prepared vector by infusion reaction. |
| 8 | pCMV-ribo-Sa-SCID-g2 | A plasmid expressing CMV promoter controlled, self-cleavable, *IL2RG*-targeting sgRNA for SaCas9. The Tetraloop of the sgRNA was replaced with com aptamer. | pCMV-ribo-sgRNA-Sa vector was cut with BsmB1 and the vector DNA was recovered; Synthesized oligo SCID-g2-Ribo-F (ccggTGTGTCCTGATGAGTCCGTGAGGACGAAACGAGTAAGCTCGTCGACACAGACAGACTACACCCA) and SCID-g2-Ribo-R (aaacTGGGTGTAGTCTGTCTGTGTCGACGAGCTTACTCGTTTCGTCCTCACGGACTCATCAGGACACA) were annealed and inserted into the BsmBI site by T4 DNA ligase. |
| 9 | pCMV-ribo-53-sp-g2 | A plasmid expressing CMV promoter controlled, self-cleavable, DMD exon 53-targeting sgRNA for SpCas9. The ST2 loop of the sgRNA was replaced with com aptamer. | pCMV-ribo-sgRNA-Sp vector was cut with BsmB1 and the vector band was recovered;  Synthesized oligo 53-sp-g2-ribo-F (CCGGaacagtCTGATGAGTCCGTGAGGACGAAACGAGTAAGCTCGTCactgttgcctccggttctga) and 53-sp-g2-ribo-R (AAACtcagaaccggaggcaacagtGACGAGCTTACTCGTTTCGTCCTCACGGACTCATCAGactgtt) were annealed and inserted into the BsmBI site by T4 DNA ligase.. |
