# Supplementary material for: Engineered extracellular vesicles as versatile ribonucleoprotein delivery vehicles for efficient and safe CRISPR genome editing
Source: J Extracell Vesicles. 2021 Mar 16;10(5):e12076. doi: 10.1002/jev2.12076 (PMC7962171; doi:10.1002/jev2.12076)
Supplement: Supplementary file 10 — Supporting Information [file JEV2-10-e12076-s001.docx]

**Supplementary Table S2. Primers Used for the study**

| Primer name | SEQ | Use |
| --- | --- | --- |
| Scid-g2F | CACCGACACAGACAGACTACACCCA | For PCR detection of *IL2RG* targeting *sgRNA*. |
| sgRNA-R3 | GATAAACACGGCATTTTGCCTTG |  |
| Reporter-F | tccatttcaggtgtcgtgag | To amplify the DNA in the GFP reporter cassette. |
| Reporter-R2 | TCCAGCTCGACCAGGATG |  |
| IL2RG-F2 | GAAGCTATGACAGAGGAAACG | Used with IL2RG-3301R to amplify the IL2RG DNA for NGS. |
| HBB-R1 | AGCCAGGGCTGGGCATAAAAG | Used to amplify the HBB region for NGS with HBB-R3 |
| HBB-R3 | TGGGAAAATAGACCAATAGGCAGAG | Used to amplify the HBB region for NGS with HBB-R or HBB-R |
| IL2RG-3301R | GGCAGCTGCAGGAATAAGAG | To amplify the endogenous IL2RG target sequence |
| DMD50-F | GCTGCTCTTTCTGGCATTG | To detect DNA deletion between sgRNA Sa-50 and DMD-53 (96kb) by PCR. |
| DMD53-R | TCCAGCCATTGTGTTGAATC |  |
| DMD53-F | TCCTGTTGTTCATCATCCTAGC | To amplify the DMD 53 exon target region for NGS |
| DMD53-R | TCCAGCCATTGTGTTGAATC |  |
| hCLCN5-F | GTTTAAGGGCCCGCCTTTTG | To amplify the CLCN5 target region for NGS |
| hCLCN5-R | TGTCTTACCTCTCGGTGCCT | To amplify the CLCN5 target region for NGS |
| DMD50-F | GCTGCTCTTTCTGGCATTG | To detect *DMD* exon 50 deletion between sgRNA Sa-50 and Sa-51 by PCR. |
| DMD51-R2 | CAGTTACAGTTATTACCGCAGCA |  |
| ABE-g5-onF | GTCTGAGGTCACACAGTGGG | For qPCR to detect base editing at ABE site 5 with ABE-g5-onF |
| g5-ABE-R | AGCCCTGACTCATCATTACCC |  |
| GAPDH-onF | AGGAGTAAGACCCCTGGACC | To amplify the GAPDH target region for NGS |
| GAPDH-onR | TCTCACCTTGACACAAGCCC |  |
| P53-onF | CTGGCATTCTGGGAGCTTCA | To amplify the TP53 target region for NGS |
| P53-onR | GAGACCTGTGGGAAGCGAAA |  |
| g5-onF | GTCTGAGGTCACACAGTGGG | To amplify the intergenic region of chromosome 20 for NGS |
| g5-onR | CTGAGAGCAGGGACCACATC |  |
