# Supplementary material for: Engineered extracellular vesicles as versatile ribonucleoprotein delivery vehicles for efficient and safe CRISPR genome editing
Source: J Extracell Vesicles. 2021 Mar 16;10(5):e12076. doi: 10.1002/jev2.12076 (PMC7962171; doi:10.1002/jev2.12076)
Supplement: Supplementary file 11 — Supporting Information [file JEV2-10-e12076-s008.docx]

**Supplementary Table S3. Target sequences and oligos for cloning guide into sgRNA-expressing vector**

| Target gene | Target sequence with PAM | sgRNA name | Forward Oligo for cloning | Reverse Oligo for cloning | Vector | Restriction enzyme |
| --- | --- | --- | --- | --- | --- | --- |
| **For SpCas9** | | | | | | |
| *IL2RG* | GCGCTTGCTCTTCATTCCCTGGG | *IL2RG* | ACCGGCGCTTGCTCTTCATTCCCT | AAACAGGGAATGAAGAGCAAGCGC | pspCas9-3'UTR-ST2-com-vector | BbsI |
| *HBB*(Sickle mutant) | GTAACGGCAGACTTCTCCTCAGG | *HBB-sp-g1* | ACCGGTAACGGCAGACTTCTCCAC | AAACGTGGAGAAGTCTGCCGTTAC | pspCas9-3'UTR-Tetra-com-vector, | BbsI |
| *DMD* Exon 53 | ACTGTTGCCTCCGGTTCTGAAGG | *DMD53* | ACCGactgttgcctccggttctga | AAACtcagaaccggaggcaacagt | pspCas9-3'UTR-ST2-com-vector | BbsI |
| CLCN5 Exon 2 | GAGGACAAGTCGTACAATGGTGG | *hCLCN5-sp-g2* | ACCGGAGGACAAGTCGTACAATGG | AAACCCATTGTACGACTTGTCCTC | pspCas9-3'UTR-Tetra-com-vector | BbsI |
| Intergenic site 5 | GATGAGATAATGATGAGTCAGGG | G5 | ACCG GATGAGATAATGATGAGTCA | aaacTGACTCATCATTATCTCATC | pspCas9-3'UTR-ST2-com-vector | BbsI |
| TP53 | CCATTGTTCAATATCGTCCGGGG | P53-g1 | ACCGCCATTGTTCAATATCGTCCG | AAACCGGACGATATTGAACAATGG | pspCas9-3'UTR-ST2-com-vector | BbsI |
| GAPDH | AGCCCCAGCAAGAGCACAAGAGG | GAPDH-g1 | ACCGAGCCCCAGCAAGAGCACAAG | AAACCTTGTGCTCTTGCTGGGGCT | pspCas9-3'UTR-ST2-com-vector | BbsI |
| **For SaCas9** | | | | | | |
| *DMD* Intron 50 | TATGTGGCTTTACCAAGGTCCCAGAGT | *Sa-50* | CACCgTATGTGGCTTTACCAAGGTCC | AAACGGACCTTGGTAAAGCCACATAc | pX601-Tetra-com-vector | BsaI |
| *DMD* Intron 51 | GTGTTATTACTTGCTACTGCA GAGAGT | *Sa-51* | CACCGTGTTATTACTTGCTACTGCA | AAACTGCAGTAGCAAGTAATAACAC | pX601-Tetra-com-vector | BsaI |
| *IL2RG* | ACACAGACAGACTACACCCAGGGAAT | *IL2RG* | CACCGACACAGACAGACTACACCCA | AAACTGGGTGTAGTCTGTCTGTGTC | pX601-Tetra-com-vector | BsaI |
